# Gesamtwirtschaftliche Stabilität im klimapolitischen Wandel

**DOI:** 10.1007/s10273-022-3154-z

**Published:** 2022-04-19

**Authors:** Willi Koll

**Affiliations:** Bonn, Deutschland

**Keywords:** E63, E64, Q58

## Abstract

Klimapolitik mit dem Ziel der CO_2_-Neutralität erfordert einen einschneidenden strukturellen Wandel. Dieser wird nur dann akzeptiert werden und gelingen, wenn er sich neben sozialer Verträglichkeit bei binnen- und außenwirtschaftlicher Stabilität vollzieht. Dafür müssen bestimmte Voraussetzungen erfüllt sein.

## Abstract (en)

Climate policy’s goal of CO2 neutrality requires drastic structural change. This will only be accepted and successful if it takes place, in addition to social compatibility, within domestic and external economic stability. Prerequisites for this are discussed in this article.

Die Klimapolitik und ihre Ziele – Klimaneutralität als Netto-Null in Europa bis 2050[1], in Deutschland bis 2045 – erfordern zusätzlich zur Digitalisierung einen lang anhaltenden Strukturwandel, weg von fossiler, d.h. $CO_2$-haltiger Produktion, Produkten und Konsum, hin zu klimaneutraler Wertschöpfung im Unternehmen und deren Verwendung.[2] Für den Erfolg der Klimapolitik sind berechenbare Vorgaben, stabile Erwartungen und eine optimale Abfolge der verschiedenen, insbesondere der be- und entlastenden Maßnahmen mitentscheidend.[3] Dieser Prozess hat eine nationale, EU-weite und globale Dimension. Im Rahmen der Klimapolitik werden insbesondere strukturelle, fiskalische und soziale Dimensionen diskutiert. Zu kurz kommt dabei der Aspekt der gesamtwirtschaftlichen Stabilität im klimapolitischen Wandel.[4] Jedoch: Ohne eine tragfähige (Makro-)Ökonomie gelingt keine nachhaltige Ökologie.

1 China und Indien haben längere Zeiträume auf der Klimakonferenz 2021 in Glasgow gefordert.
2 Eine Fokussierung auf Klimapolitik muss dennoch die gesamte Breite der Umweltpolitik im Blick behalten. Auf die Aufzählung der Vielzahl an Maßnahmen wird verzichtet. Der Offene Brief eines Dutzends Wissenschaftlicher Beiräte der Bundesregierung nennt „den zügigen Ausbau der erneuerbaren Energien, einen schnellen Infrastrukturausbau für Energiewende und klimafreundliche Mobilität, den Einstieg in eine Wasserstoff-Ökonomie, den effektiven Schutz der biologischen Vielfalt, eine konsequente Reduzierung des Flächenverbrauchs, den Aufbau einer Kreislaufwirtschaft sowie eine Transformation der Agrar- und Ernährungssysteme“ (Rat für Nachhaltigkeit, 2021; auch Bundesministerium für Wirtschaft und Klimaschutz, 2022).
3 Unsicherheit wirkt wie eine zusätzliche Steuer auf Investitionsfähigkeit und -bereitschaft (Brunnermeier, 2022).

**Dr. Willi Koll** war Ministerialdirigent in den Grundsatzabteilungen des Bundeswirtschafts- und -finanzministeriums. Zugleich war er Mitglied im Wirtschaftspolitischen Ausschuss der EU und der OECD sowie im Makroökonomischen Dialog der EU.

## Struktureller Wandel

Der strukturell erforderliche Wandel benötigt die Bepreisung fossiler und die Förderung klimaneutraler Produktion und Verwendung. Diese „pretiale“ Lenkung macht fossile Produktion und Produkte progressiv teurer. Die Nachfrage sinkt entsprechend der Preiselastizität.[5] Solange die Preiselastizität der Nachfrage nach fossilen Gütern gering bleibt und gleichzeitig die Grenzkosten ihrer Vermeidung steigen, bedarf es umso stärkerer Preissteigerungen, um den gewünschten Mengeneffekt über Umstrukturierung, höhere Energieeffizienz oder Einsparung zu erzielen. Der fossile Kapitalstock wird dabei zunehmend entwertet und abgeschrieben. Gleichzeitig müssen Subventionen und Privilegien für fossile Produktion und Produkte abgebaut werden ebenso wie Belastungen klimaschonender Wertschöpfung. Bei zugleich steigender Nachfrage nach klimaschonenden Produkten werden in der privaten Wirtschaft neue Investitionen und ihre Finanzierung erforderlich und rentabel.[6] Die erforderliche Umstrukturierung erfordert parallel eine komplementäre, klimaschonende öffentliche Infrastruktur, insbesondere im ÖPNV und im Netzausbau.[7] Dadurch sinkt der verbleibende Anpassungsbedarf, der über Bepreisung erfolgen muss, entsprechend.

4 Auch der jüngste Jahreswirtschaftsbericht der Bundesregierung geht hierauf nicht ein (Pisani-Ferry, 2022).
5 Die Preiselastizität der Nachfrage nach fossilen Gütern wird für private Haushalte schwerpunktmäßig kurzfristig auf weniger als -0,25, langfristig auf weniger als -0,5 geschätzt, für Verkehr auf -0,25 bzw. -0,5 (Bach et al., 2019, 6 ff.).
6 Soweit die Vermeidungskosten noch deutlich über dem $CO_2$-Preis liegen, werden Carbon Contracts for Difference (CCfD) vorgeschlagen (Verband der Chemischen Industrie, 2020).
7 Der weltweite Investitionsbedarf zur Erreichung der Klimaziele wird mit über 100 Bio. US-$ veranschlagt (SVR, 2021, Tab. 24, 397).

## Gesamtwirtschaftliche Entwicklung

Für den Erfolg des klimapolitischen Strukturwandels, seiner sozialpolitischen Flankierung und seiner gesellschaftlichen Akzeptanz ist eine nachhaltig günstige gesamtwirtschaftliche Entwicklung bei hoher Beschäftigung und gutem Einkommen eine notwendige Bedingung.[8] Im Ergebnis ändert sich dann durch Klimapolitik die Struktur, nicht aber das Niveau der gesamtwirtschaftlichen Entwicklung. Ein hoher Beschäftigungsstand setzt insbesondere ein konfliktfreies Zusammenwirken von Geldpolitik sowie Lohn- und Preisentwicklung voraus, unterstützt soweit erforderlich durch eine stabilisierende Fiskalpolitik (Koll, 1988, 57 ff.). Unter dieser Voraussetzung können Arbeitsplätze, die im strukturellen Wandel wegfallen, zahlenmäßig an anderer Stelle neu entstehen. Ein geeignetes Gremium zur Abstimmung der wirtschaftspolitischen Akteure kann der Makroökonomische Dialog auf EU-/WWU und nationaler Ebene sein (Koll und Watt, 2022).

## Stabile Lohn-, Preis- und Einkommensentwicklung

Voraussetzung für eine stabile gesamtwirtschaftliche Entwicklung ist, dass die Sozialpartner die Verteuerung fossiler Produkte in ihrer Wirkung auf die Gewinne der Unternehmen und die Kaufkraft der Arbeitskräfte hinnehmen und nicht flächendeckend als Zweitrundeneffekt in Löhne und Preise überwälzen, sondern sich bei den Lohnabschlüssen weiterhin am Produktivitätszuwachs plus Preisstabilitätsziel der Geldpolitik orientieren.[9] Andernfalls würde aus einer singulären, wenn auch breiten Preissteigerung eine flächendeckende Inflation.

Jedoch absorbiert die $CO_2$-Bepreisung einen Teil der nominalen Kaufkraft. Um weiterhin die bisherigen nichtfossilen Güter und Dienstleistungen absetzen und kaufen zu können, müssten deren Preise entsprechend sinken. Wenn dafür Gewinne und Löhne abgesenkt würden, würde das wiederum die verbliebene Kaufkraft senken und ist insofern keine Lösung. Nur bei einer (unverzögert wirksamen) Preiselastizität der Nachfrage nach fossilen Gütern von -1 bleiben nominale Kaufkraft und reale gesamtwirtschaftliche Nachfrage erhalten, damit auch Produktion und Beschäftigung, aber auch die Akzeptanz einer stabilitätsorientierten Lohn- und Preisentwicklung und letztlich auch des strukturellen Wandels. Ist die Elastizität niedriger als 1, muss der Kaufkraftverlust ausgeglichen werden, um den bisherigen gesamtwirtschaftlichen Warenkorb ohne Preissenkung kaufen zu können. Hierfür muss das Aufkommen aus der Bepreisung fossiler Produkte in den Wirtschaftskreislauf zurückgeschleust werden, sei es in Form eines direkten Transfers an die privaten Haushalte, deren Entlastung an anderer Stelle oder als öffentliche Infrastrukturinvestitionen bzw. Förderung privater Investitionen klimaschonender Art.

Gleichzeitig entsteht zum einen ein klimapolitisch verursachter höherer Abschreibungsbedarf; dieser verringert ceteris paribus in den Volkswirtschaftlichen Gesamtrechnungen das Wachstum der Produktivität pro Kopf und damit des Verteilungsspielraums; zum anderen wird das verbleibende Produktionspotenzial stärker für klimaschonendere Investitionen in Unternehmen und öffentliche Infrastruktur verwendet. Bei Vollauslastung der Kapazitäten bedeutet das einen relativ geringeren Anteil an Konsumgüterproduktion.[10] Bei gleichbleibendem nominalem Einkommen würde es daher aufgrund eines geringeren gesamtwirtschaftlichen Warenkorbs an „konventionellen" Gütern und Dienstleistungen zu einer temporären Preisanspannung kommen. Diese wirkt aber zusammen mit der $CO_2$-Bepreisung wiederum positiv auf klimaschonende Investitionen, Innovationen und technischen Fortschritt. Die damit verbundene Ausweitung des Produktionspotenzials führt ihrerseits wieder zu einer Entspannung bei der Preisentwicklung.

Die Umstrukturierung des Kapitalstocks muss einhergehen mit der Umschulung von Arbeitskräften. Je flexibler die betriebliche Struktur bei Human- und Sachkapital, umso stärker können der strukturelle Wandel innerbetrieblich erfolgen und ganze Schließungen vermieden werden. Soweit es hier zeitlich zu Friktionen zwischen wegfallenden und neuen Arbeitsplätzen kommt, müssen diese durch arbeitsmarkt- und sozialpolitische Maßnahmen überbrückt werden.[11] Bei qualitativen Friktionen muss aktive Bildungs- und Weiterbildungspolitik, gegebenenfalls unter Verkürzung der Arbeitszeit, für einen Übergang sorgen.

## Rolle der Geldpolitik

Die Geldpolitik ist durch den strukturellen Wandel neben makroprudenziellen Risiken vor allem konjunkturell gefordert. Makroprudenziell geht es dabei um die Stabilität von Finanzmärkten und Bankensektor, konkret um die

8 Die Nichterfüllung dieser Bedingung wird zu Recht als belastender angesehen als die Verteuerung fossiler Güter (Dullien und Stein, 2022).

9 “If they [central banks] assess it to be short-lived and unlikely to affect the medium-term inflation outlook, they may ‘look through’ such a shock. Under these conditions, the central bank may tolerate the temporary effects on inflation without taking any action, in order not to cause undue volatility in output and employment. If they assess the shock to be more persistent, with a risk that it may lead to second-round effects on wages and inflation and an unanchoring of inflation expectations, monetary policy action may be warranted” (ECB, 2021, 122).

10 Pisani-Ferry (2021, 9) zieht hier eine Parallele zu Militärausgaben.

11 Der Sachverständigenrat erwog vor Jahrzehnten, das Tempo für die Einführung verschärfter Umweltschutzbestimmungen zu verringern. „Weichere Standards dürfen jedoch nur für eine kurze Anpassungsdauer gewährt werden. Es macht gesamtwirtschaftlich keinen Sinn, die internationale Konkurrenzfähigkeit von Branchen und die Erhaltung unrentabler Arbeitsplätze in umweltintensiv produzierenden Bereichen mit Umweltschäden zu bezahlen“ (SVR, 1984, Ziff. 408).

### Kasten 1
### So nicht: Ölpreiskrisen 1973 und 1979

Die beiden Ölpreiskrisen des vergangenen Jahrhunderts haben gelehrt, wie es nicht gehen darf, hier aufgezeigt am Beispiel der ersten Krise. Der Bepreisung von fossilen Gütern entsprach damals die schockartige Anhebung des Ölpreises auf rund das Fünffache. Von der vorhandenen Kaufkraft musste ein erheblich größerer Teil an die ölexportierenden Länder abgegeben werden. Verstärkte Käufe der Ölländer in den ölimportierenden Staaten führten zwar einen Teil der entzogenen Nachfrage zurück. Es blieb jedoch der Entzug von eigener Kaufkraft; die Terms of Trade und damit die Einkommensverteilung hatten sich zugunsten der OPEC-Staaten verschoben. Die Sozialpartner überwälzten den Anstieg der Ölpreise auf Löhne und Preise. Im Zeitraum 1974/1975 belief sich der jahresdurchschnittliche Anstieg der Bruttolöhne pro Kopf auf rund 12 %, der Lohnstückkosten auf 8 % und des BIP-Deflators auf gut 6 % (Bundesministerium für Wirtschaft, 1975, 33). Die Geldpolitik, durch die vorangegangene Freigabe der Wechselkurse nun autonom, reagierte auf diese erste Drehung der Preis-Lohn-Spirale mit mehr als einer Verdopplung der Bundesbankzinsen und nahezu einer Halbierung der Wachstumsrate der Zentralbankgeldmenge (Deutsche Bundesbank, 1974, 14 und 5). Wachstum und Beschäftigung brachen aufgrund der Kostenexplosion und Nachfrageimplosion drastisch ein; die Arbeitslosigkeit stieg erstmalig schockartig an. Bei allen Unterschieden zwischen Ölpreisexplosion und $CO_2$-Bepreisung geschah im Ergebnis das Gegenteil dessen, was eine makroökonomisch verträgliche Klimapolitik ausmacht.

Werthaltigkeit von Vermögensgegenständen und um die Risikoeinschätzung. Die Bepreisung von fossiler Produktion verändert über eine abnehmende interne Rentabilität insgesamt den Börsen- und Marktwert von Unternehmen und speziell den Wert des Anlagevermögens. Soweit dieses als Besicherung von Krediten dient, ändert sich deren Abdeckungsgrad. Im Extremfall müssen Vermögensgegenstände total abgeschrieben werden (*stranded assets*). Diese Effekte beeinflussen im Urteil der Geldpolitik den Transmissionsmechanismus bei der Wahrung der Preisstabilität.[12]

Konjunkturell wird die Preisstabilität durch die Preiseffekte der Klimapolitik tangiert. Die klimapolitisch notwendige permanente Verteuerung fossiler Produkte schlägt sich mit deren Gewicht im Harmonisierten Verbraucherpreisindex (HVPI) nieder. Bei einer unverzögert wirksamen Preiselastizität der Nachfrage bei fossilen Gütern von -1 bleibt die gesamte Preisentwicklung unverändert.[13] Liegt die Elastizität dagegen deutlich unter -1, steigt das Preisniveau aufgrund des steigenden Anteils der fossilen Güter daran stetig an. Unter den beschriebenen lohn- und preispolitischen Voraussetzungen ist das jedoch immer noch keine Inflation, sondern Ergebnis einer Verteuerung zwar vieler, aber immer noch nur einzelner, hier fossiler Güter. Wird das Aufkommen aus der $CO_2$-Bepreisung in Form einer Klimaprämie den privaten Haushalten zurückerstattet, muss dieser Preiseffekt bei der geldpolitischen Beurteilung der Preisentwicklung aus dem HVPI herausgerechnet werden (HVPIX, d. h. HVPI-„ex $CO_2$“). Die Geldpolitik müsste diesen $CO_2$-bedingten Preissteigerungseffekt tolerieren und entsprechend kommunizieren.[14] Ein solches Verhalten der Geldpolitik lässt sich vermeiden, wenn dieser Preiseffekt an anderer Stelle neutralisiert wird. Hierzu bietet sich eine aufkommensneutrale Senkung anderer beim Verbrauch anfallender Abgaben an. Ein ergiebiger Kandidat ist hier die Mehrwertsteuer.

## Die europäische Dimension

Auf europäischer Ebene findet derzeit eine Überprüfung des wirtschaftspolitischen Rahmens (*economic governance review*) statt. Sie fokussiert primär auf Ersatz oder Flexibilisierung des Stabilitäts- und Wachstumspakts (SWP). Was den Klimaschutz betrifft, wird bei der Flexibilisierung des SWP unter anderem die Einführung einer Green Golden Rule diskutiert, die grüne öffentliche Investitionen von der Defizitregel ausnehmen und deren Umfang weitgefasst werden soll. Die bereits verabschiedete Aufbau- und Resilienzfazilität (ARF) enthält klare Anforderungen an den Anteil an klimapolitischen Ausgaben der nationalen Aufbau- und Resilienzpläne.

Was die gesamtwirtschaftliche Entwicklung betrifft, hat es auf EU-Ebene, insbesondere der Wirtschafts- und Währungsunion (WWU), einen Konflikt zwischen Geldpolitik und aggregierter Lohn- und Preisentwicklung wie in Deutschland bei den Ölpreiskrisen 1973 und 1979 bisher nicht gegeben (vgl. Kasten 1). Dafür haben sich, auch ohne dass schon ein massiver klimapolitischer Strukturwandel die wirtschaftliche Entwicklung belastet hätte, nach dem Beginn der WWU kumulativ Divergenzen zwischen den Mitgliedstaaten entwickelt, die 2010 in eine schockartige

12 “... climate risks may affect the transmission of monetary policy through financial markets and the banking sector, notably via the stranding of assets and sudden repricing of climate-related financial risks” (ECB, 2021, 108).

13 Der Anteil der fossilen Güter an der gesamtwirtschaftlichen Preisentwicklung bleibt dann konstant.

14 “The impact on inflation and output growth depends on how the central bank reacts. Choosing to look through the impact of the relative price shift and target core inflation results in a limited impact on headline inflation and a slightly negative impact on output .... Targeting headline inflation results in a lower inflationary impact overall, at the expense of a greater negative impact on GDP.” (ECB, 2021, 14).

Krise des Euroraums mündeten. Um so viel mehr noch muss deshalb im Zuge des klimapolitischen Strukturwandels darauf geachtet werden, dass sich diese Divergenzen nicht wiederholen und so nicht erneut krisenhafte Probleme für die makroökomische Entwicklung hinzukommen.

Hierfür ist eine Reform des Verfahrens zur Vermeidung und Korrektur makroökonomischer Ungleichgewichte (Macroeconomic Imbalances Procedure, MIP) im Rahmen der derzeitigen Überprüfung des wirtschaftspolitischen Rahmenwerks der EU erforderlich. Insbesondere muss das MIP-Verfahren in seinen Indikatoren und Schwellenwerten symmetrisch, ökonomisch aussagekräftiger sowie politisch beeinflussbar umgestaltet werden. Wie im nationalen Kontext ist im Rahmen des MIP vor allem darauf zu achten, dass sich Lohnstückkosten und Preisentwicklung aller Mitgliedstaaten am Preisstabilitätsziel der EZB orientieren (Koll und Watt, 2022).

## Klimapolitik im außenwirtschaftlichen Rahmen

Klimawandel ist eine global wirkende Entwicklung. Optimal ist daher ein global wirkender Strukturwandel,[15] moderiert auf globaler Ebene, ausgehend von einem $CO_2$-Restbudget, heruntergebrochen auf nationale $CO_2$-Budgets (Wiegand et al., 2021, 133; Mazzucato, 2021) und fair verteilt zwischen den Generationen (Bundesverfassungsgericht, 2021). Präferenzen, Ausgangssituation, Mittelausstattung und Inzidenz unterscheiden sich jedoch gravierend innerhalb der Weltgemeinschaft, insbesondere zwischen Industrieländern einerseits und Schwellen- und Entwicklungsländern andererseits, aber auch zwischen den jeweiligen Sektoren (Vergleich zu Abrüstungsverhandlungen: Koll, 1990, 24). Das gilt vor allem auch für die Verursachung des Klimawandels – historisch, aktuell und zukünftig. Ein weltweit gleichzeitiger und gleichstarker struktureller Wandel ist daher nicht zu erwarten.

Solange das nicht der Fall ist, haben Länder, die fossile Güter mit einer Steuer, Zertifikaten oder Regulierung belegen, unter sonst gleichen Bedingungen einen Nachteil im preislichen Wettbewerb. Das Ergebnis wäre unter anderem eine Passivierungstendenz der Leistungsbilanz und die Verlagerung umweltbelastender Produktion ins Ausland (direktes Carbon Leakage) (SVR, 2021, Plustext 13). Zum Ausgleich dieses Nachteils wird vorgeschlagen, wenngleich kontrovers diskutiert, die Importe mit einem Zoll (Carbon Border Adjustment Mechanism, CBAM) zu belegen. Zugleich müssten fossilhaltige Exporte entsprechend entlastet werden.[16]

15 Die Klimakonferenz 2021 in Glasgow war ein Schritt in diese Richtung.

16 Die Einführung von CBAM wird derzeit in der EU verhandelt. Eine derartige Neutralisierung der außenwirtschaftlichen Effekte durch Exportsubventionen und Importzölle war schon vor 40 Jahren vorgeschlagen worden (Flassbeck und Maier-Rigaud, 1982, 44, FN 65). Einen Anhaltspunkt für diesen Ausgleich liefert die EU-Agrarpolitik mit ihren Erstattungen und Abschöpfungen seit Jahrzehnten.

Ein weltweiter Mindestpreis für $CO_2$ könnte die notwendige Intensität der Maßnahmen abmildern.

Eine theoretische Alternative ist die Absenkung bzw. Mindersteigerung des gesamtwirtschaftlichen Lohn- und Preisniveaus im Maße der durchschnittlichen umweltpolitisch verursachten gesamtwirtschaftlichen Verteuerung zur Erhaltung der preislichen Wettbewerbsfähigkeit. Branchen mit unterdurchschnittlicher $CO_2$-Intensität würden dann sogar in der grenzüberschreitenden preislichen Nachfrage profitieren, solche mit überdurchschnittlichem $CO_2$-Anteil entsprechend per Saldo belastet bleiben. Diese Maßnahme wäre außenwirtschaftlich hilfreich, binnenwirtschaftlich aber eher problematisch (Keynes, 1973, 267). Ähnliche Effekte hätten Subventionen für inländische Produkte und Investitionen zur Verringerung des $CO_2$-Gehalts, jedoch sind hier international wettbewerbsverzerrende Effekte nicht auszuschließen (Petersen, 2021, 349). Alternativ wird als $CO_2$-Bepreisung eine binnenwirtschaftliche Verbrauchsabgabe entsprechend dem $CO_2$-Gehalt aller Produkte vorgeschlagen. Diese fiele dann für relativ stärker $CO_2$-belastete Importe umso höher aus (Petersen, 2021, 349).[17]

Schließen sich Länder mit gleichen Umweltabgaben zu einem Klimaklub nach Nordhaus zusammen, entfällt der Ausgleich innerhalb dieser Länder; ein gemeinsamer Grenzausgleich wird dann gegenüber dem Rest der Welt wirksam. In einen solchen Klub könnten auch Schwellen- und Entwicklungsländer aufgenommen werden und ein Lastenausgleich intern erfolgen (SVR, 2021, Ziff. 628). Ein solcher Klub könnte die EU über den Green Deal werden.[18] Er wäre groß genug, den Strukturwandel nicht nur wenigen Staaten zu überlassen, einen entsprechend stärkeren Klimaeffekt zu erzielen, vor allem eine technologische Pionier- und Vorreiterrolle zu übernehmen und Anreiz zur Nachahmung zu geben.[19] Die Erwartung ist, dass diesem Klimaklub möglichst schnell und zahlreich weitere Länder beitreten, wenn und weil auch für sie eine aktive Klimapolitik unabweisbar wird. Eine Vorreiterrolle wäre dann im internationalen Preiswettbewerb neutralisiert; güterwirtschaftlich kann sie als positiver Wettbewerbsfaktor angesehen werden.[20]

17 Ein Grenzausgleich eines größeren Klimaclubs wird dabei gegenüber einer Verbrauchsabgabe vorgezogen (Wissenschaftlicher Beirat beim Bundesministerium für Wirtschaft und Energie, 2021).

18 Die EU hat hierzu ein Gesetzespaket verabschiedet, das derzeit im Europäischen Parlament und Ministerrat verhandelt wird. Hierfür müssen neben dem bestehenden EU-weiten Emissionshandel für Stromerzeugung und einigen Industriebereichen auch die nationalen Handelssysteme für Wärme und Verkehr EU-weit ausgestaltet werden.

19 Zwar ist die EU für weniger als ein Zehntel der Treibhausgase verantwortlich, aber „… die annähernde Aufrechterhaltung bzw. die Wiederherstellung einer intakten globalen Umwelt wird nur gelingen, wenn die ärmeren Länder in ihrem Aufholprozess in den reichen Ländern ein umweltpolitisches Vorbild finden“ (Koll, 1996, 197).

20 „Ich bin davon überzeugt, dass wir uns mit klimaschonenden Lösungen deutlich von Konkurrenten abheben können…“ Brzoska (2021).

Im Gleichschritt mit der Reduzierung der (globalen) Nachfrage nach fossilen Gütern muss ihr (globales) Angebot reduziert werden. Andernfalls sinken bei unverändertem Angebot aufgrund der Mindernachfrage des Klimaklubs die Preise für fossile Güter und das umso mehr, wenn die Ölländer ihr Angebot daraufhin noch ausweiten (Grünes Paradoxon) (Sinn, 2008). Das wiederum regt eine Mehrnachfrage der Nicht-Klimaklub-Länder an und vermindert ihren Anreiz zum strukturellen Wandel (indirektes Carbon Leakage) (SVR, 2021, Ziff. 535; Flassbeck, 2021, 91 ff.). Andererseits könnte eine gemessen an der Nachfrage überschießende Verknappung beim Angebot den Marktpreis fossiler Güter so erhöhen, dass er annähernd auf dem klimapolitisch vorgesehenen Preispfad verläuft und für eine $CO_2$-Besteuerung und eine Rückerstattung an die inländischen Haushalte weder Bedarf noch Möglichkeit bleiben.

Spätestens in dem Maß, wie sich Klimaclubs ausweiten und damit trotz Mehrnachfrage des verbleibenden aber abnehmenden Rests der Welt die Gesamtnachfrage sinkt, dürften die Gewinnerwartungen der fossilen Anbieter ebenfalls sinken und Anreiz geben, alternative Geschäftsfelder zu erschließen. Auch die Förderländer sollen insofern in internationale Abkommen einbezogen werden (SVR, 2021, 531 und 536).

## Sozialer Ausgleich

Klimawandel trifft Menschen mit niedrigem Einkommen stärker als solche, die sich aufgrund höheren Einkommens und Vermögens den Folgen (zunächst) leichter entziehen können. Deshalb ist eine aktive Klimapolitik gerade mit Blick auf die Schwächsten notwendig. Aber auch der Strukturwandel und insbesondere die damit verbundenen Abgaben belasten die Schwächeren relativ stärker als Reiche (Dullien und Stein, 2022). Um diesen Effekt zu kompensieren, muss die Regressivität der Klimamaßnahmen über Rückerstattungen oder Entlastungen neutralisiert werden.

Wie zuvor angesprochen gibt es rein technisch mehrere Möglichkeiten der Rückerstattung. Zum einen könnte sie über eine Kopfpauschale als Klimaprämie erfolgen. Zum anderen könnte die Abgabenbelastung an anderer Stelle kompensatorisch und aufkommensneutral abgesenkt werden. Vorschläge hierzu liegen in Form einer Senkung der Stromsteuer oder der Abschaffung der EEG-Umlage vor. Diese Maßnahmen würden bürokratische Probleme wie bei der Klimaprämie vermeiden. Angesichts der absehbar massiven Erhöhung der $CO_2$-Abgaben zur Erreichung der Klimaneutralität dürfte das Aufkommen von Stromsteuer und EEG-Umlage für einen vollen, längerfristigen Ausgleich nicht ausreichen. Dann bedarf es der Senkung einer ergiebigeren Steuer. Hierfür bietet sich die Mehrwertsteuer an. Eine Klimapauschale als Kopfpauschale geht dabei sogar über eine volle Kompensation bei unterdurchschnittlichem Verbrauch hinaus, im Gegensatz zu einer Mehrwertsteuersenkung, die nur am tatsächlichen Konsum anknüpft. Jedoch sollte zum einen Umverteilung nicht über Klimapolitik angestrebt werden; zum anderen überwiegen die Vorteile einer aufkommensneutralen Steuersenkung insbesondere mit Blick auf Preisentwicklung und Geldpolitik bei weitem. Dies setzt voraus, dass funktionierender Wettbewerb die Weitergabe der MwSt-Senkung gewährleistet.

Ein Ausgleich für klimapolitische Entlastungen darf sich nicht auf die nationale Volkswirtschaft beschränken. Er muss sich auch auf das Einkommensgefälle zwischen Industrie- und Schwellen-, bzw. Entwicklungsländern erstrecken. Das ist nach dem Verursachungsprinzip, der Tragfähigkeit, aber vor allem auch für das Gelingen des notwendigen weltweiten Klimaschutzes erforderlich. Wo bisher elementare Bedürfnisse nur um den Preis von Umweltzerstörung und $CO_2$-Freisetzung befriedigt werden können, müssen andere umweltschonende Möglichkeiten geschaffen werden. Eine Anpassung der Bedürfnisse an die der Industrieländer sollte dabei deren umweltpolitische Fehler nicht wiederholen, sondern überspringen, unter anderem durch Technologietransfer, angepasste Handelsabkommen und internationale Klimafinanzierung (SVR, 2021, Ziff. 559 ff.).[21] Aber gerade auch in den Schwellen- und Entwicklungsländern sind für Akzeptanz und Realisierung einer effektiven Klimapolitik eine stabile gesamtwirtschaftliche Entwicklung, ausreichende Arbeitsplätze und eine gute allgemeine Politik notwendig. Ein leistungsfähiges Bildungssystem sollte dazu beitragen, auch das Bevölkerungswachstum in nachhaltige Bahnen zu lenken.

## Schlussbemerkung

Die Erkenntnis in die Notwendigkeit einer forcierten Umwelt- und Klimapolitik war schon vor Jahrzehnten vorhanden. Das Gleiche gilt für ihre makroökonomisch verträgliche Gestaltung.[22] Ebenso lange wird ein Paradigmenwechsel hin zu nachhaltiger Entwicklung, Regeneration, Revidierbarkeit von Eingriffen, Eingriffsminimierung und intertemporärer Substanzerhaltung gefordert (z. B. Klawitter, et al., 1990, 200; Maier-Rigaud, 1997, 339). Aber ebenso lange gab es nur ein nahezu „folgenloses Erken-

21 Die Klimafinanzierung für Entwicklungsländer sollte als fester Anteil aus dem Aufkommen aus der $CO_2$-Bepreisung in den Industrieländern fixiert werden. Zusätzlich sollten letztere die Multilateralen Entwicklungsbanken mit ausreichend Kapital ausgestattet werden, um ein Vielfaches an Krediten vergeben zu können (Sachs, 2021).

22 Schon Anfang der 1980er Jahre gab es viel Literatur zur Thematik (insbesondere Flassbeck und Maier-Rigaud, 1982; SVR, 1984, Ziff. 400 ff.). Im Lauf der Jahrzehnte hat sich diese Agenda vom breiten Umweltschutz auf den Klimawandel fokussiert.

nen“ (Maier-Rigaud, 1988, 113 ff.; Wicke, 1991, 107).[23] Erst die Massierung von klimabedingt eingetretenen und unmittelbar absehbaren Katastrophen hat seit Kurzem einen Wandel im Denken erzwungen. Folgt dem neuen Denken nun auch ein starkes Handeln?[24] Eine hinreichende Antwort auf diese vielschichtigen Fragen kann die Ökonomie alleine nicht geben. Hier bedarf es ihres Zusammenwirkens mit vielen anderen wissenschaftlichen Disziplinen wie z. B. Naturwissenschaft, Individual- und Sozialpsychologie, Politologie, Bildungs- und Kommunikationswissenschaft, insbesondere im Bereich der sozialen Medien (Klawitter et al., 1990). Jedoch: Ohne eine tragfähige Ökonomie gelingt keine nachhaltige Ökologie – allerdings gilt je länger, desto mehr auch die Umkehrung dieser These.

23 „Doch die Weltgemeinschaft weigert sich seit 30 Jahren beharrlich, aus den Erkenntnissen der diesjährigen Physik-Nobelpreisträger [Klaus Hasselmann und Syukuro Manabe] ebenjenen Nutzen zu ziehen“ (Wiedlich, 2021, 1).

24 Dabei gibt es nach wie vor Lücken zwischen dem, was nötig und dem, was beschlossen ist, z. B. zwischen einem politischen Ziel von 1,5 °C Erwärmung und einer Politik, die mit derzeitigen und geplanten Maßnahmen gerade einmal 2,4 °C, mit Unterfütterung dieser Pläne mit kurzfristigen Zusagen und Maßnahmen 1,8 °C erreichen wird (Höhne, 2021, 2). Bei einem Verharren auf dem derzeitigen Emissionsplateau wird sogar mit 3,5 °C gerechnet (Mann, 2021, 15).

**Title:** ***Macroeconomic Stability in the Climate Policy Transition***
